# Dual Effect of the Cubic Ag_3_PO_4_ Crystal on *Pseudomonas syringae* Growth and Plant Immunity

**DOI:** 10.5423/PPJ.NT.09.2015.0191

**Published:** 2016-04-01

**Authors:** Mi Kyung Kim, Byul-Ee Yeo, Heonyong Park, Young-Duk Huh, Chian Kwon, Hye Sup Yun

**Affiliations:** 1 Department of Molecular Biology, Dankook University, Yongin 16890, Korea; 2 Department of Chemistry, Dankook University, Yongin 16890, Korea; 3 Department of Biological Sciences, Konkuk University, Seoul 05029, Korea

**Keywords:** cubic silver phosphate crystal, plant immunity, *Pseudomonas syringae*, silver

## Abstract

We previously found that the antibacterial activity of silver phosphate crystals on *Escherichia coli* depends on their structure. We here show that the cubic form of silver phosphate crystal (SPC) can also be applied to inhibit the growth of a plant-pathogenic *Pseudomonas syringae* bacterium. SPC pretreatment resulted in reduced *in planta* multiplication of *P. syringae*. Induced expression of a plant defense marker gene *PR1* by SPC alone is suggestive of its additional plant immunity-stimulating activity. Since SPC can simultaneously inhibit *P. syringae* growth and induce plant defense responses, it might be used as a more effective plant disease-controlling agent.

Since antiquity, silver due to its antibacterial activity has been diversely used in commercial products and medical applications (Klaine et al., 2008). Recently, it has been revealed that the silver ion (Ag^+^) can induce the production of reactive oxygen species (ROS) by disrupting metabolic pathways and increases membrane permeability by inhibiting disulfide bond formation in bacteria, which leads to bacterial cell death (Morones-Ramirez et al., 2013). However, due to the higher cytotoxicity of Ag^+^, particle-embedded silvers are preferentially studied and used as antibacterial agents.

Effect of silver either as an ionic or a nano-sized particle form has been also investigated in plants. It has been reported that both Ag^+^ and silver nanoparticles (AgNPs) have a contrasting effect on plant growth: stimulatory at low concentration but inhibitory at high concentration (Vannini et al., 2014; Wang et al., 2013). Transcriptomic and proteomic analyses by Ag^+^ and AgNP have revealed that they mostly control the expression of stress-related genes (Kaveh et al., 2013; Vannini et al., 2014). However, the presence of non-overlapping genes changed by Ag^+^ and AgNP indicates that nanoparticle itself used to encage silver has its own specific effect on plants (Kaveh et al., 2013; Vannini et al., 2013; Vannini et al., 2014). We previously found that the cubic silver phosphate (Ag_3_PO_4_) crystal (SPC) with six facets had a greater effect on inhibiting *Escherichia coli* growth than the rhombic dodecahedral one with twelve facets (Yeo et al., 2015). Since any particle effect can be ruled out by using SPC, we therefore tested whether or not it could be applied for controlling a plant disease caused by phytopathogenic bacteria.

We synthesized SPC and checked its structure by powder X-ray diffraction and scanning electron microscopy (SEM) as described previously (Fig. 1A, *inset*) (Yeo et al., 2015). We then started to grow the Arabidopsis-pathogenic *Pseudomonas syringae* DC3000 bacterium with 0.06 Absorbance at 600 nm (A_600_) in the presence or absence of SPC. *P. syringae* DC3000 grown at 28°C without SPC almost reached plateau at 1 d post inoculation (Fig. 1A). However, at the same time point, we detected no significant bacterial growth in the presence of SPC (Fig. 1A). Although bacterial growth with 0.5 μg/ml of SPC reached the comparable level to that without SPC after a longer incubation period (6 d post inoculation), bacteria with 5 μg/ml of SPC completely failed to grow (Fig. 1A). To further test the effect of SPC on bacterial growth, we additionally counted bacterial numbers by counting colonies in the growth medium containing or not SPC. Similar to results obtained by measuring optical density (Fig. 1A), numbers of bacteria in the presence of SPC were significantly reduced compared to those without SPC (Fig. 1B). Interestingly, we still detected bacterial colonies even in the presence of 5 μg/ml of SPC with which bacterial population never increased (Fig. 1B). This implies that SPC suppresses bacterial cell division rather than kills bacteria. Taken together, our data indicate that as in the case of *E. coli* SPC can also effectively inhibit the growth of phytopathogenic *P. syringae* DC3000 bacterium. This also suggests a potential agrochemical activity of SPC to control a bacterial disease in plants.

To test whether SPC has any additional disease-controlling activity in plants, we then examined bacterial growth inside plant tissues. We first treated 10 d-old Arabidopsis plants grown in liquid medium with different amounts of SPC for 2 d and liquid-inoculated those plants with *P. syringae* DC3000. To exclude the direct growth-inhibiting effect of SPC on *P. syringae* DC3000, SPC-treated plants were extensively washed to remove surface SPC and transferred to new liquid medium followed by bacterial inoculation. At 3 d post inoculation, we found that bacterial growth was significantly reduced in plants pretreated with 5 and 50 μg/ml of SPC compared to that in non-treated plants (Fig. 2A). This suggests that SPC may directly increase plant immune responses to diminish bacterial multiplication. Therefore, we then investigated the expression of *PR1* which is the best representative marker gene of elevated plant immunity. In plants treated with 50 μg/ml of SPC, we found that the expression of *PR1* was enhanced in a time-dependent fashion compared to non-treated ones (Fig. 2B). Since simple mechanical stress by solid SPC can affect the expression of *PR1*, we additionally examined the expression of *CAF1a* whose transcription is induced by mechanical wounding (Walley et al., 2010). However, we found that unlike *PR1* the expression of *CAF1a* was not increased by SPC (Fig. 2C). This indicates that SPC has an additional role in specifically boosting plant immune responses.

In summary, we here show that SPC has a dual activity to inhibit the growth of a plant-pathogenic *P. syringae* DC3000 and to elevate plant immune responses. It is not surprising that SPC could affect the growth of *P. syringae* DC3000, because we previously found that it inhibited the growth of *E. coli*. Our previous analysis on SPC structure suggested that Ag^+^ in the cubic form is better outward exposed than the rhombic dodecahedral one (Yeo et al., 2015). Hence, the increased exposure of Ag^+^ in the cubic SPC is thought to inhibit the growth of *P. syringae* DC3000 as well as *E. coli*. However, the plant immunity-elevating activity of SPC was not expected. To rule out a direct bactericidal activity of remaining SPC itself, we washed treated plants. In addition, the side length of SPC that we synthesized is between 1.5 and 2.5 μm (Fig. 1A, *inset*), which makes it difficult to enter plant leaf tissue via stomata. More importantly, *PR1* expression was dramatically elevated in plants treated with SPC only (Fig. 2B). Although an exact mechanism to induce plant immune responses by SPC is currently unknown, one possibility is that Ag^+^ can induce ROS generation by disrupting metabolic pathways in plants as in bacteria (Morones-Ramirez et al., 2013). It has been reported that the plant immune hormone salicylic acid (SA) and ROS form a positive feedback loop in plant immunity (Bartsch et al., 2006). Since SPC is not likely to enter plant tissues due to its size, SPC-induced ROS generated from epidermal cells is suggested to stimulate the SA-associated immune responses in plants. Although we extensively washed SPC-treated plants, it is still possible that a small fraction of treated SPC remains on leaf surface. Since we liquid-inoculated plants with bacteria, therefore, it cannot be ruled out that remained SPC inhibits at least in part the epiphytic bacterial growth.

## Figures and Tables

**Fig. 1:**
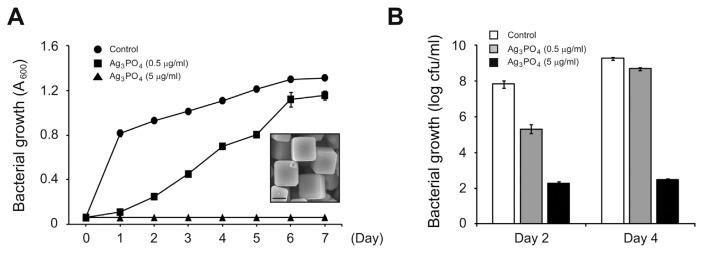
Inhibition of *P. syringae* DC3000 growth by SPC. Overnight-grown bacteria were diluted and aliquoted into new tubes with A_600_ = 0.06. They were further grown with or without the indicated amounts of SPC. Bacterial growth was then analyzed at the indicated time points by measuring values of A_600_ (A) or by counting bacterial colonies (B). Data represent mean±SD from three biological replications. (*inset* in A) The cubic structure of synthesized SPC was analyzed by SEM. Bar, 1 μm.

**Fig. 2:**
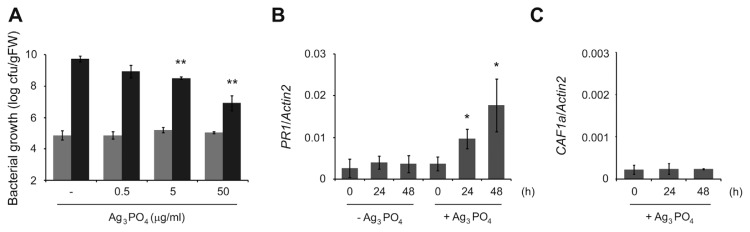
Induction of plant immune responses by SPC. (A) SPC inhibits the *in planta* growth of *P. syringae* DC3000 in a dose-dependent manner. Ten day-old plants were treated with the indicated amounts of SPC for 2 d and liquid-inoculated with *P. syringae* DC3000 with A_600_ = 0.02. At 4 h (grey bar) or 3 d (black bar) after inoculation, bacterial numbers inside plants were counted. (B and C) SPC induces the expression of *PR1* (B) but not *CAF1a* (C). Ten day-old plants were treated with or without 50 μg/ml SPC for the indicated time. RNA extracts were then subject to realtime RT-PCR analysis with a LightCycler^®^ Nano System (Roche). Primers used were; 5′-TTCTTCCCTCGAAAGCTCAA and 5′-AAGGCCCACCAGAGTGTATG for *PR1*, 5′-TGATGTTAACGGTAACCTCCCAGAC and 5′-GCCGACGAAGCAACTCGATCGAATC for *CAF1a*, and 5′-ATGGAATCTGCTGGAATCCAC and 5′-TTTGCTCATACGGTCAGCGAT for *Actin2*. Relative transcript amounts of *PR1* and *CAF1a* were normalized against *Actin2*. Bar, mean±SD from three biological replications. *, *P*<0.05; **, *P*<0.01 in comparison to non-treated plants.
